# Illustrations of the Efficacy of Compression and Percussion in the Cure of Rheumatism and Sprains, Scrofulous Affections of the Joints and Spine, Chronic Pains Arising from a Scrofulous Taint in the Constitution, Lameness, and Loss of Power in the Hands from Gout, Paralytic Debility of the Extremities, General Derangement of the Nervous System; and in Promoting Digestion, with All the Secretions and Excretions

**Published:** 1824-06

**Authors:** William Balfour

**Affiliations:** the University of Edinburgh.


### ART. II.—*Illustrations of the Efficacy of Compression and Percussion in the Cure of Rheumatism and Sprains, Scrofulous Affections of the Joints and Spine, chronic Pains arising from a Scrofulous Taint in the Constitution, Lameness, and Loss of Power in the Hands from Gout, Paralytic Debility of the Extremities, General Derangement of the Nervous System; and in promoting Digestion, with all the Secretions and Excretions.* By WILLIAM BALFOUR, M.D. of the University of Edinburgh.

ALL skilful and conscientious physicians, says Dr. Gregory, will have recourse, in all cases, to those remedies which have been found, from *indubitable experience*, to be useful, whether their mode of operation is understood or not; and will abstain from those which the same experience has proved to be useless or hurtful. For the science of medicine is beneficial so far only as it leads to the discovery of efficacious remedies, and a successful method of cure. Nor is it, indeed, consistent either with reason or common sense to confide in theoretical opinions, however specious, which are contrary to experience."*

Now, though the practice illustrated in the following cases rests on the immoveable foundation of "indubitable experience," which Dr. Gregory, and every other man of common sense, says is paramount to all theory—to all reasoning; yet I will not concede that the mode of operation of compression and percussion, in the complaints enumerated in the heading of this paper, is either inexplicable or unintelligible. It is equally obvious with that of the drawing of a tooth, in curing tooth-ache,—with that of the local detraction of blood, in effecting the resolution of local inflammation,—and infinitely more explicable and intelligible than the mode of operation of mercury, in curing syphilis, or that of cinchona, in curing intermittent fever. But, having in a former work explained the principles on which, I apprehend, compression and percussion prove efficacious remedies, in those diseases in which I have employed them, I confine myself at present to a statement of facts, not more extraordinary than incontrovertible.

It has been objected, that other practitioners do not obtain the results from compression and percussion which I have ascribed to them: it would be a *miracle* if they did. But the objection, while it proclaims the originality of the practice, has really no foundation whatever. In the first place, I apply the remedy *with my own hands*, leisurely and perseveringly; other practitioners satisfy themselves with *prescribing* it, without once touching their patients, and then persuade themselves that they have done justice to my practice, and their duty to their patients? But, as a knowledge of anatomy, physiology, and pathology, are as necessary to the efficacious application of compression and percussion as in any other species of practice, medical or surgical, a practitioner would, with the same pro priety, tell a patient in a phrensy to treat himself so and so, as he would merely *prescribe* compression and percussion in such cases as are hereafter detailed. What respect, then, is due to the candour of those who have so little respect for themselves as to deny a practice of which they never made trial?

In the second place, it will be admitted, I presume, that, *cæteris paribus*, any individual practitioner is likely to excel in the treatment of a disease to which he has long particularly directed his attention, and in which his experience is extensive. Now, I originated and introduced the practice of compression and percussion, in the cure of rheumatism and complaints allied to it; I have devoted a great share of my attention to it for ten years, with increasing success; and I have now brought it to a

* *Conspect. Medicin. Theoret.* 964.

degree of perfection almost incredible to those who are not acquainted with the history of the cases which have been cured by it, after every other method had been tried in vain. This is no vain boast or arrogant assertion. The thing can be proved by evidence, the most ample and unexceptionable. It is not common sense to suppose that ladies and gentlemen would come from every county in Scotland, from England, from Ireland, to consult me, were they not previously assured of the astonishing success of the mode of treatment I have introduced, and practise: it is not common sense to suppose that so many of the poor would apply to me, after having attended the Royal Infirmary and Dispensaries here in vain, did they not derive that relief from my mode of treatment, which they can obtain from no other.

But I must not be misunderstood. When I say that patients of the highest rank come to me from afar,—that patients apply to me after having been long under medical treatment, in vain, —I intend no disrespect to any practitioner whatever, but the contrary. I mean neither more nor less than that compression and percussion cure many cases of rheumatism, and complaints allied to it, over which medicine, directed by the most eminent skill, has no power. If in this there is disrespect to any human being, then I know not the import of the terms respect and disrespect;—if in this there is egotism or presumption, then no practitioner is at liberty to promulgate any discovery he may make, though it were to save the human race from perdition;—if a practitioner is to suffer reproach for stating the most undeniable facts, because they redound to his credit, then farewel to every honourable and legitimate motive to exertion in the cause of medical science and humanity. How can mankind be benefited by any improvement, if they are not made acquainted with it? How can any new practice be appreciated, but by a comparison of its effects with those of other modes of cure which have preceded it?

Besides removing pain, swelling, and rigidity of the joints, muscles, and other parts, compression and percussion exert a powerful and salutary influence on all the functions of the body, vital, natural, animal; and, therefore, promote general health in a remarkable degree. That such must be the case, is evident from the following considerations:

When the pulse is preternaturally frequent, as in acute rheumatism, the operation of compression and percussion diminishes its frequency, in a very palpable degree. I have brought it down six, ten, and fourteen strokes in a minute. (Case 27, &c.)

When the pulse is hard, small, and wiry, or irregular, compression and percussion render it soft, full, and regular. (Case 30, &c.)

On the brain and nerves, compression and percussion produce the most powerful effects. (Cases 37 and 38.) See the note to Case 38, which illustrates the subject most satisfactorily. Such, indeed, is the efficacy of percussion in promoting an equal distribution of the nervous power, that I am strongly inclined to believe it would be found a powerful remedy in tetanus. That it would be an efficient auxiliary to the other means employed in suspended animation, there cannot be a doubt. Nothing could so readily excite the action of the brain and nerves,—nothing could so immediately and powerfully impart motion to the blood.

On the digestive organs, compression and percussion exert a powerful and salutary influence. I have often been told by my patients, without their being questioned on the subject, that they felt an unusual craving for food immediately after an operation. One lady (Case 24,) told me she was obliged to take a lunch every day on which she had an operation. Another (Case 31,) declared she was compelled to eat immediately after an operation. A late learned lord, whose life, according to his own spontaneous acknowledgments, was prolonged for many months by the operation in question, after Dr. Gregory ceased to prescribe for him, ate voraciously every day after the operation; whereas, he was frequently overpowered with sickness previous to its application.

Intestinal excretion is greatly promoted by the application of compression and percussion to the trunk of the body, but particularly the abdomen. (Case 40.) An elderly gentleman felt himself unwell, but could give no other account than that his bowels, which were irregular, would not answer to medicine, and that his feelings were uneasy. He consulted various practitioners of the highest respectability, successively, without benefit. Compression and percussion, particularly to the abdomen, were at last resorted to, with perfect success: his bowels became regular without the aid of medicine, and his uneasy sensations disappeared.

In patients who have long laboured under rheumatism or scrofulous affections of the joints, and consequently have been deprived of that exercise in the open air which is necessary to perfect health, I have often observed the skin to have a morbid appearance, (Cases 29 and 38,) and to be dry, rough, and rigid. These effects are owing to deficiency of nourishment. But compression and percussion, by promoting circulation in the parts, produce all the good effects of voluntary motion or exercise. The limb, which was wasted, recovers flesh; the skin acquires a healthy appearance, and becomes smooth and flexible.

Should it be asked by the professional reader, what I would do in cases where there is too much, or inflammatory, action?

I would refer him to Cases 29, 30, &c. I would also request him to consult a paper of mine on *Whitlow*, in vol. xxxix. of this Journal, page 185. There he will find that the practice I am advocating is available, even where the highest inflammatory action prevails. In one word, compression and percussion moderate the action of the system, or of parts, when in a state of preternatural excitement; but prove tonic and stimulant to the system, or to parts, in a state of atony. This may appear paradoxical at first view, but is not more so than that tonic and stimulant medicines should prove sedative in certain conditions of the body.

CASE I.—David Leitch, aged thirty, came under my care on the 4th October, 1817: had been affected with rheumatism over all his body for eighteen months, which deprived him of the power of following his occupation, that of a shawl-weaver; had been several weeks in the Royal Infirmary, and afterwards humanely attended by one of the surgeons of that institution, in his own lodgings, without deriving the smallest benefit from either. When he applied to me, he was reduced to skin and bone, and could neither rise up, nor sit down, nor walk, but with the greatest difficulty and pain. His appearance was that of an aged man, nearly famished. Though the whole body was more or less affected, yet pain and rigidity were greatest in the back, loins, hips, hip-joints, and thighs.

From such a distressful condition was this young man rescued in a month,—not by the power of medicine, for that had no control over the disease; otherwise he would not have been dismissed, in the circumstances described, from the Royal Infirmary, where there is always a concentration of professional talent and ability, surpassed in no similar institution under the sun. The cure was effected solely by the simple operation of compression and percussion. The patient gained flesh and strength rapidly; was at his loom in six weeks; and has continued well ever since, a period now of six years and a half.

CASE II.—Mrs. M'Grigor, aged thirty-five, applied to me in May, 1820. She had lost the use of her left arm, from rheumatism in the shoulder-joint, for nine years. Travelling with her husband, who was in the militia, she had opportunity of consulting various practitioners; from none of whom, however, did she derive the smallest benefit, the affection having resisted every application. At last she was persuaded that dislocation had taken place; and accordingly had, for many years, allowed the limb to take care of itself, and to dangle by her side when she walked, as if paralytic. When the arm was allowed to hang by her side, she had little or no pain; but, on attempting to raise it, the pain was excruciating, and extended backwards to the shoulder-blade. The bones grated harshly when the limb was moved; and all round the joint, but particularly on the front of it, the slightest compression occasioned painful crepitation, or a crackling noise.

This woman did not apply to me in the hope of being benefited by my exertions, but purely in compliance with the injunctions of a lady of distinction, who patronised her. But, though convinced that nothing could do her any good, she found herself, at the end of two weeks, capable of washing her family's clothes. In the course of six weeks, pain and crepitation were entirely removed, and the natural motions and command of the arm completely restored. In these circumstances the patient went to the country, and I know nothing farther of her history.

CASE III.—William Young, a sailor, thirty years of age, was recommended to me, in the summer of 1820, by one of the gentlemen of the Trinity-house, Leith, in very distressing circumstances. He had been rendered incapable of doing any thing for his own support for three years and a half, from rheumatism in his right arm. In the course of his illness, he had been thirteen weeks in the Royal Infirmary, without being in the least benefited; and was afterwards confined to his bed for twelve months. He was now begging his bread.

The disease was seated in the shoulder-joint, elbow-joint, and wrist; with an oblong tumor on one of the bones of the fore-arm, as hard to the touch as bone. All the parts affected were most excruciatingly painful, on the slightest touch or motion; so painful, indeed, that any other practitioner, less accustomed to such cases, would have considered the application of compression and percussion altogether inadmissible, and calculated rather to excite pain and inflammation than cure the disease. Nevertheless, by compression and percussion alone was this young man perfectly cured, in the short space of eleven days; and he immediately procured employment at Leith, in the profession to which he was bred. I never saw this young man again; but heard of him being well twelve months afterwards, through the medium of other distressed paupers, who applied to me from his recommendation.

CASE IV.—A lady, fifty-two years of age, consulted me, in July 1820, for a rheumatic affection of her left knee-joint, of great severity and long standing. There was great swelling all round the joint, with considerable effusion; and the limb, having been long kept in a half-bent posture, could not now be extended. The pain stretched from the knee, along the muscles, to the very top of the thigh; so that, from pain, the position of the joint, and want of command of the moving powers of the limb, the patient was incapable of walking. This rendered a weakly constitution still more delicate; nor did she ever expect to be again able to take exercise on foot in the open air. At my first visit, indeed, she politely assured me that, should I fail to do her good, she would not be disappointed, or reflect on me, as she had already had the best skill, and perseveringly tried every thing that could be thought of, in vain.

But, maugre this lady's despair of her condition ever being ameliorated, I succeeded in a short time in removing the pain, and in extending the limb completely; so that she has ever since been able to take as much exercise as she pleased, and her health is long ago perfectly restored. This lady could take no medicines.

CASE V.—A gentleman from the south of England, about fifty years of age, put himself under my care, in February 1821. His left arm was affected with rheumatism, accompanied by a painful tension of the nerves, from the top of the shoulder to the first joint of the thumb inclusive. But, though the limb was generally affected, the pain was greatest behind the shoulder, at the bend of the arm, on the outside and a little below the flexure of the joint, where the bones are thinly covered, in the deep-seated muscles of the fore-arm, and about the root of the thumb. When the arm was extended, the nerves running along the inside were rendered so painful, by being put upon the stretch, as to prevent rotation of the fore-arm; and the outside of the elbow-joint was so painful to the touch, that the patient, in walking the streets, was in continual terror and alarm, lest any person would come within a foot of it, being ready to faint from the slightest rub. An aching pain also often prevented rest in the night-time.

In this condition had the arm been for eighteen years, in spite of every thing that could be done in London and elsewhere. So much, indeed, had been done in vain, that the patient and his friends had long despaired of ever obtaining a cure. Without the aid, however, of a particle of medicine, either externally or internally, a perfect cure was effected in twenty-eight days, by compression and percussion. This gentleman had also long laboured under lumbago, which was completely removed at the same time, and by the same means.

CASE VI.—H. M., a young woman, twenty-six years of age, came from Lanarkshire, in the latter end of July, 1820, to the Royal Infirmary of Edinburgh. Twelve months before, she had been seized with a rheumatic fever of great severity; the consequences of which were, pain and weakness in the loins, the hips and hip-joints, and a slight curvature of the lumbar vertebræ, with tension or contraction of both sciatic nerves and their branches. The affection in the loins gave her a stooping posture, and the contraction of the nerves in the back of the thighs prevented a full extension of the limbs. In one word, when placed against a perpendicular wall, if her head was made to touch it, her heels could not be brought within a foot of it. Conversely, if her heels were put in contact with the wall, her head could not be brought within a foot of it.

The medical treatment she had received from the beginning of her illness, was in strict conformity with the established practice of the day. A caustic issue had been made in each hip, below and behind the trochanter major, and kept open for a great length of time, without producing the least benefit. When, therefore, the patient told her story to the physicians and surgeons of the Royal Infirmary, they informed her they could do nothing more for her than follow on the treatment she had already been under. "Then," said she, "I may as well go home, and continue under my own surgeon?" "You certainly may," was the reply. Accordingly, she forthwith left the house, but was met in the suburbs by an army-surgeon, (the late Archibald Hamilton, esq. fourteen years surgeon in the 92d regiment,) who knew some of her connexions, and whom I cured of a painful rheumatic affection in one of his wrists, of three years and a half's standing. He strenuously advised the patient to return, and put herself under my care; which she did, and was perfectly cured of pain and deformity in three months.

CASE VII.—James Smith, aged thirty-five, had complained, for sixteen months, of pain and weakness in his loins, accompanied with general debility. In the course of his illness, he had abundance of medical aid, in the Royal Infirmary and from other quarters, but without the slightest advantage. When he applied to me, in June 1820, he was so weak and emaciated, that he could not walk a quarter of a mile; and the pain in the loins, and contracted state of the sciatic nerve and its branches, rendered him incapable of stooping. He could go up a stair, but could not go down, any jolting motion occasioning great pain in the loins.

I commenced my treatment of this case by the application of percussion over the connexion of the last lumbar vertebra with the sacrum, and the first blow, a gentle tap with my hand, occasioned a tingling sensation in the loins internally, and sent a rush of nervous power down the back of the thigh and leg, to the very heel. When percussion was applied to the superior lumbar vertebræ, no sensation was produced; but, applied to the upper part of the sacrum, and below the brim of the pelvis, a slight sensation was produced, if great force was used. From these data I concluded that the trunk common to the fourth and fifth lumbar and the three superior sacral, which unite to form the sciatic, were the nerves chiefly affected. This, indeed, was put beyond a doubt, from the fact of the sensation produced by the operation being confined to the parts supplied by the sciatic nerve and its ramifications.

It may seem surprising that the mechanical power of percussion should induce such a salutary change in nerves in a state of morbid sensibility and tension; but there is no resisting, or contending with, the evidence of facts. In the present case, immediately after the first operation, which was not continued more than ten minutes, the patient stooped a foot nearer the ground than what he could do before I handled him. Next day, he walked up a very long stair, got an operation, and then went down without inconvenience. The operations were continued every day for six weeks, when the patient could walk six miles without resting; and in two months was, and has continued ever since, at his usual employment.

This was one of those cases in which the beneficial effects of percussion on the general health were very apparent. When the man applied to me, he had the appearance of one far gone in consumption, but was not a fortnight under the operation when his countenance began to clear up, his spirits became buoyant, his body agile, and he felt the beginning of returning strength. Two gentlemen, whom he had successively served as coachman, and who took an interest in him, as being a decent man, and having a young family to provide for, called on me, after he had been a few days under my care, with the avowed purpose of seeing what sort of a being I was, who could produce such effects in so short a time, and by means so simple. I mention the fact as a proof of the patient's own account, to his old masters and benefactors, of the benefit he had derived, and was deriving at the time, from the operations.

Case VIII.—Mr. A. N., aged seventy, had for a long period been lame, and had suffered excruciating pain, from a rheumatic affection in the right hip, extending round the hip-joint into the groin, and down as far as the knee. All the usual remedies had been tried for years, in vain; and the parts were now so tender as to require the most delicate management.

The slightest percussion produced a vibration in the nerves, as if a shock of electricity had been communicated. But, notwithstanding the great age of the patient, the inveteracy and severity of the disease, compression and percussion, applied at first very gently, and gradually with more freedom, effected a perfect cure in three weeks. So completely, indeed, were the tenderness and pain of the parts removed, that the old man could bear, without flinching, a blow of my hand, inflicted with my utmost force.

CASE IX.—Mr. W. M., from Fifeshire, aged fifty-two, consulted me on the 13th of July, 1820, for an affection of the right hip-joint, hip, and thigh, of five years and a half's standing. The limb was an inch shorter than the other, rotation of the thigh was very obscure, and flexion and extension were also extremely confined. In going up or down a stair, he always advanced the left foot, and brought up the right alongside of it. In walking on a plain, he also stepped short with the right foot, not being able to bring the heel to the point of the left great toe. The whole hip was very much pained, but certain spots about the front of the hip-joint, and about the outside and top of the thigh, he could scarcely suffer to be touched. He did not complain of these parts, however, except when they were handled: on the contrary, he was astonished when I discovered to him that his disease was so widely extended. The only pain he had in moving the limb, or when passive motion was given to it, was in the knee; a symptom that, combined with the diseased state of the brim of the acetabulum, proves the complaint to have been as much of a scrofulous as of a rheumatic nature.

By compression and percussion, alternated with passive motion of the limb on the hip-joint, was this gentleman, in less than a month, not only freed from pain, but enabled to walk with perfect freedom, even up and down a stair. He could advance the right foot equally with the left, and plant it upon a chair; whereas, when he applied to me, he could not, as has already been observed, bring the right heel to the point of the left great toe. The limb was also perceptibly extended in length. To my great satisfaction, I heard, two years afterwards, that this gentleman continued to enjoy all the advantages he had derived from the operations. Indeed, I have had several other patients from his neighbourhood, merely through his representations of the benefit he experienced.

CASE X.—Walter Nesbit, aged twenty-six, of a full habit of body, called on me, on the 24th July, 1820, in the greatest possible agony, from a rheumatic affection in the back of the right thigh, running along the whole course of the sciatic nerve. The complaint had existed only a short time, but attacked in paroxysms of intolerable severity. He was in one of these as he entered my house, but I checked it in less than a minute by forcible compression. I now made the patient himself practise compression in my presence, in order to make sure of his being able to coerce the paroxysms in my absence. A perfect and permanent cure was effected in two days.

CASE XI.—A young woman sprained one of her ankles in the harvest of 1821 so violently, that she could not suffer her foot to touch the ground. In that condition she was brought to me in a cart, a distance of forty miles, in the full conviction that her labours were at an end for the season. By the application of compression and percussion, however, she was enabled to walk in two days, and forthwith returned to her work.

Case XII.—On the 2d July, 1820, J. F., aged thirty, consulted me for rheumatism in his knees, accompanied with a considerable degree of fever. He came in a hackney-coach, was literally lifted out, and entered my house supporting himself on a staff, and more than half carried by a person on each side of him; his knees refusing to carry his weight, and bending so far forward as to prevent an erect posture. Both knees were pained, but one of them was also considerably swelled and inflamed, particularly round the base of the patella. Pulse 90, and hard; tongue white. Complaint had existed only two or three days.

After expressing my astonishment that the patient should have come out in such a condition, instead of sending for me, I proceeded to the application of compression and percussion to the parts affected. This I did with the greatest caution and gentleness at first, but gradually with more freedom, as the parts became soothed. At intervals I also gave passive motion to the joints. In this way I continued the operation for an hour, when flexion and extension of the joints were perfectly restored, and the patient walked away without support of any kind, carrying his staff horizontally, in order to show the perfect freedom of motion he had acquired. Before he rose, I examined the pulse again, which one would have expected would now have been more frequent; but, instead of that being the case, it was softer, and six strokes in a minute less frequent than when I began the operation.

In this case I combined antimonial medicines with the operations, and with the very best effects. I visited the patient, and went through the operations, on the two succeeding days; when he set out for London by sea, much against my advice. He returned in less than three weeks —not well, but not worse; and four or five more operations completed the cure.

Case XIII.—Mr. J. O., a young gentleman, consulted me, in 1821, for a rheumatic affection in and about the tendon of the right heel, of three years and a half's standing. At times he felt little inconvenience, comparatively; at other times he was very lame. These alternations were influenced by the state of the weather, and the patient's own conduct; but the complaint, true to its nature, became gradually more fixed and severe. This gentleman, like too many others, believed there was no cure for rheumatism but "patience and flannel," and for a long time regarded my mode of treating the disease as an illusion. An accumulation of undeniable facts, however, induced him at last to submit to compression and percussion, whereby he obtained a perfect cure in less than a week. If this gentleman carried his scepticism an unjustifiable length at first, he afterwards made ample amends by the publicity he gave to his case, from a principle, I believe, of unaffected philanthropy.

Case XIV.—Before the termination of the late war, a serjeant of artillery, whose name has escaped me, had been invalided on account of rheumatism in his legs and arms. He retired with his family to Edinburgh, where he had abundance of medical aid from practitioners of the highest eminence, but without deriving the slightest advantage from the remedies prescribed. His was a case, indeed, in which medicine was of no avail; otherwise, he could not have failed to obtain benefit from the skill of the individuals who advised him. At last he applied to me, after being reduced to skin and bone; his legs, from pain and weakness, unable to support his body, and his arms incapable of the least exertion.

In a fortnight, the skin, from being of a dingy yellow, began to assume a healthy appearance; pain was removed from the legs and arms; and, being a weaver, the man was at his loom in three weeks. Had the practice I am illustrating been known and followed in the army, there had been no occasion for invaliding this man, who was at the time under forty years of age: he would not, indeed, have been off duty a single day. I am warranted in this observation, from the fact, that, after labouring under disease for a number of years, by which his health and strength, to say nothing of the pain he suffered, were completely exhausted, he was cured in three weeks. I am not singular in thinking that, were my practice adopted in the army and navy, it would prove a great public benefit: officers of high rank, in both services, have spontaneously declared their opinion to the same effect, and have urged me to memorialise government on the subject.

Case XV.—A gentleman, aged thirty-six, consulted me on the 16th April, 1821, for rheumatism in the inside of his right thigh, a little above the knee. At first the pain was trifling, but had gradually increased to such a degree as to render the patient quite lame, and the parts almost unsusceptible of being touched. Great general debility; pulse 130; total want of rest in the night-time, as well from general uneasiness of feeling, as from the local pain. Had been under the care of an old, able, and experienced physician, for a considerable length of time, but without any good effect.

In such circumstances, I applied compression and percussion with the utmost gentleness and caution, but continued the operation for a considerable length of time; when the patient declared that the pain was greatly diminished, and that all his feelings were soothed. Two more operations removed the pain entirely.

I heard no more of this gentleman till the 4th of June following, when I was called to visit him at his own residence, a considerable distance from Edinburgh. He now informed me that, after the third operation, on the 18th of April, he felt himself quite relieved, both from local pain and general uneasiness; that he dashed at once into his usual excessive action, of superintending concerns so various and distant, as would have afforded sufficient employment to three or four men instead of one; and that one evening, after a day of incredible fatigue, he was exposed to a bleak, piercing wind, during a ride of sixteen miles, which completely knocked him up.

His nerves now seemed quite unhinged, his constitution shattered to pieces; his pulse ranged from 130 to 133, small and tremulous; he could not sleep; he could neither lie nor sit in any posture for ten minutes together. I considered him dying of fatigue. There was a spot about the middle of the right leg, outside the skin, between the tibia and fibula, so exquisitely painful, that the patient referred to it as the cause of all his distress, if not of his exhaustion. There was no external appearance of disease in the part; but, when touched, the most excruciating pain shot from it in every direction. Notwithstanding, I again had reconrse to compression and percussion; but applied them, at first, at a distance from the affected part, gradually and cautiously approaching it as the patient's feelings permitted me. In half an hour, he could suffer it to be handled with freedom, and all his feelings were so soothed, that he lay on a sofa in perfect ease, and conversed cheerfully as long as I remained in the house, and, as I was afterwards informed, for the remainder of the evening. Having advised an antimonial medicine to be taken three or four times a-day, and an effervescing draught when he felt hot and restless, I took my leave, without being desired to repeat my visit, as the family-surgeon was in attendance; but rather, I believe, from the patient's imagining my simple operations might be as well performed by others as by me. But he reckoned without his host, for I was recalled on the 29th, and found him as ill as ever. I now continued to visit him every second or third day, till the 27th of August, when the pain in his leg and his uneasy feelings were entirely and permanently removed. Sleep had also returned, and he had nothing to complain of but debility. At every visit I applied compression and percussion, with the same soothing effects as at first. Without the operations, indeed, his existence could not have been prolonged. They never failed to remove pain, general uneasiness, and irritation; and though their effects, at first, were only temporary, yet they gave respites from suffering, and gradually induced that salutary change in the state of the nervous system which I have stated. With the exception of effervescing draughts, the patient would take no medicine. His food consisted chiefly of animal jelly, grapes, and a little wine. I saw this gentleman in Edinburgh in the depth of winter following, engaged in business, and apparently in good health.

Case XVI.—George Hunter, aged seventy, a poor man, applied to me on the 3d of July, 1820, for a painful affection, or rheumatism, as he called it, in his left inferior extremity, reaching from the top of the haunch to the ankle. He had been long very lame, and incapable of working. The whole hip was affected, and the pain took a direction down the outside of the thigh, then penetrated between the heads of the leg-bones, where it was most severe, and gradually became more superficial as it approached the ankle.

This man lived two miles off, but came to me almost every day, and frequently in a cart, being unable to walk so far, from mere pain and weakness in the limb. The parts affected were extremely tender to the touch, and the slightest degree of percussion caused a vibration in the nerves from the top of the thigh to the ankle. The complaint was soon removed, however, from all the parts above the knee; but, from between the heads of the tibia and fibula, it could not be dislodged but by forcible percussion, repeated almost every day for two months. Not but that the patient felt relief from every operation; otherwise I would not have continued it, nor he his attendance. Yet it was very difficult to give permanency to its effects, on account of the deep seat of the disease, its inveteracy, and the great age of the patient. A cure, however, was effected,—not partial, but complete and permanent. I have often seen this man since, walking as well as ever he did in his life, and working for his bread.

CASE XVII.—William Brown, aged forty-five, applied to me on the 26th of June, 1820, for rheumatism in his loins, right hip, and thigh. The complaint had existed a long time, but had increased during the last three months, so as to render him incapable of working. He could not bend his body forward, and the whole limb was extremely weak, and perceptibly smaller than the other. He had used liniments and swallowed medicines to a great extent, without the slightest benefit.

Compression and percussion soon removed pain and stiffness from the loins, but there was a single point in the under and back part of the thigh, (between the tuber ischii and trochanter major, but nearer the former than the latter,) which constrained the patient in all his motions, especially in stooping, and was so deeply seated as to be almost beyond the reach even of percussion. I succeeded, however, in removing the rigidity and pain of the nerves in this spot also, by putting the sciatic nerve and its ramifications upon the stretch, and then applying percussion forcibly. A complete and permanent cure was effected in a month, and the patient has been free from complaint ever since.

CASE XVIII.—On the 7th November, 1821, I was called to a young gentleman, an officer in the army, lately returned from the north, where he had been to enjoy himself in the moors during the autumn, when he was attacked with rheumatism in his right elbow-joint, which put a stop to his amusement. The affection had existed several weeks before I saw him, during which he had suffered the greatest agony. All the usual remedies, particularly fomentations, had been employed with great perseverance, but without material advantage. The joints and neighbouring parts were now inflamed, tense, and swollen; and a deep-seated throbbing pain kept the patient in such a state of irritation, that he got no rest night or day. Pulse 96.

It will not, perhaps, be anticipated by the professional reader, that I would employ compression and percussion in a case of this nature. It may even be considered a case in which such a mode of cure is inadmissible. Nevertheless, I have to state that I did employ compression and percussion, once every day, and sometimes twice, with the most soothing effects; and, at the same time, gave passive flexion and extension to the joint, and rotation to the fore-arm, that I might not have a stiff, or perhaps an immovable, joint to contend with, after inflammatory action was subdued. It must not be imagined, however, that, in conducting these operations, I used force: they were conducted in the slowest, gentlest, and most cautious manner, entirely under the regulation of the patient's feelings. While this was going on, I administered emetic tartar also, with great freedom and effect: not, however, in nauseating doses, for then the patient would never have taken a second, but in such quantity as his stomach could easily bear. In this way the medicine could be, and was often, repeated; and produced all the sedative effects that could be desired, without the slightest distress. A perfect and permanent cure was effected in three weeks, and the motions of the joint and fore-arm preserved entire.

CASE XIX.—A young lady, from the west of Scotland, when on a visit to her friends in the south of England, in the summer of 1821, sprained one of her ankles severely. Pain, puffiness of the part, and difficulty in making any great exertion in walking, continued for three months. In this state she arrived in Edinburgh, in the end of November. About the beginning of December, she was seized one night, on going to bed, with increased pain in the ankle, and with rheumatism in the shoulder and knee of the same side, which completely prevented sleep the whole night, and she rose in the morning in great pain.

Making her situation known to the family in which she resided, my services were immediately required. The affection of the shoulder and knee was severe, but, being recent, were removed in one day. Not so the pain in the ankle, which was extremely acute, and required the utmost delicacy in being handled. Notwithstanding, my fair patient declared she felt sensible relief from every operation, and the cure was complete in four days from the time I was called in.

CASE XX.—On the 10th December, 1821, John Kirkup, a stout young man, a painter, employed at the new College, fell from a height of two stories, on the pavement. He alighted on his feet, and immediately fell prostrate. Finding he was not materially hurt in the trunk of his body, he attempted to rise, but his feet were incapable of performing their functions. He was carried home, and I saw him about twelve o'clock, an hour after the accident. Considerable swelling had already taken place, particularly about the inside of the arches of both feet; one heel was much bruised, and the tendons of the toes were violently sprained; the other foot was injured chiefly about the outer ankle, and the insertion of the tendon of the heel.

I desired the patient to show me how he could stand and walk. He insisted he could do neither; but, upon my pressing him, attempted to get up, and fell back again on his bed, complaining grievously of the excruciating pain the effort occasioned. I made this experiment that he might be able to form a just estimate of the effects of the operation I was about to perform on him. I now went over all the injured parts, applying compression with my hands slowly and gently, and then percussion. These were continued, under the regulation of the patient's feelings, for nearly half an hour, and were succeeded by bandages. I now desired him to stand up, and try to walk. He did both, though with considerable pain. The same operation was performed in the evening, twice next day also, and once on the day following. Pain and swelling were sensibly diminished by every operation. When I called on him on Thursday, precisely three days after the accident, I found him putting on his usual shoes, for the purpose, he said, of coming to me. He afterwards admitted he was down stairs the preceding day, walking in the street. After this he had only one operation, when he returned to his work.—This and Case XIth. demonstrate, I think, what compression and percussion can do, when timely applied to parts that have suffered violence.

Case XXI.—On the evening of the 26th December, 1821, I was seized all at once, about ten o'clock, with a shivering fit. I instantly had percussion applied over the shoulders, true ribs, and spine, which immediately checked the febrile paroxysm. The night was wet, and the streets very dirty, and I had been visiting from seven till ten. For some days previously I felt pains shooting through my whole body, and the day before had had a sort of diarrhœa. On going to bed, I had another fit of shivering, and a renewal of the diarrhœa, which continued through the whole night. In the morning I experienced most oppressive head-ache; my pulse was about a hundred, and the motion of the heart so strong that I felt not only my body, but the bed, shaken by every stroke. I sent for a medical friend, who prescribed bleeding in the arm, and the application of a dozen of leeches to the temples. I absolutely felt as if my head was about to separate in the direction of the coronal suture. I thought my friend's advice very rational, but had a strong desire to try emetic tartar previously, thinking I could do no less than treat myself as I treated others. Accordingly, I took sixty drops of antimonial wine in some boiled rice and milk, with sensible relief to the head-ache; and had percussion sedulously applied to the inferior extremities, where I experienced violent shooting pains. I repeated the emetic tartar several times, in smaller doses, in the course of the day, with increased good effects, both as to the head-ache and the force of the heart. At bed-time, having still some remains of head-ache, I took sixty drops of antimonial wine, as in the morning. In about half an hour I became sick, and disposed to vomit. This sensation lasted a moment only, but recurred twice afterwards at short intervals, and with as short duration. I did not vomit, but the dejections, which had become less frequent, were renewed. Instead of being aqueous, however, they were now fæcal, of greater consistence, and occurred only twice. Within an hour from taking the last dose of the medicine, I was as free from complaint as ever I was in my life. The oppression I had laboured under was entirely removed, my spirits became buoyant, my strength returned, the diarrhœa left me, and I slept soundly the whole night. Next day I went abroad, and visited my patients as usual. I had a severe attack of rheumatism again in my left knee, but removed it entirely in the course of the day by repeated applications of percussion.

Case XXII.—A tradesman's wife, aged thirty, consulted me, about the middle of December, 1821, for rheumatism, with which she had been afflicted for two years, without intermission. The whole of the right side of the neck and shoulder were most excruciatingly pained; so much so that at first she could scarcely suffer them to be touched, and therefore concluded they were incurable. It was in consequence, indeed, of her husband witnessing the effects of my operations on John Kirkup that she was induced to apply to me. The left elbow-joint was also much pained, and permanently bent; both wrists were painful and enlarged; and several of her fingers were also so painful and swollen at the joints, as to be incapable of flexion and extension. On the whole the patient was rendered unfit for the performance of her domestic occupations, except very imperfectly and with torture; circumstances certainly, to a woman in her situation, sufficiently distressing.

This woman called on me only five times altogether, and these visits were all made in the course of a fortnight. To her astonishment, the pain in the neck and shoulder, which she was persuaded could never be removed, was scarcely felt at the fourth operation. The elbow-joint was considerably more extended, and less pained; the swelling and pain of the wrists were diminished, and she could shut her hands with facility and firmness. She complained, however, that, though her wrists and fingers were in a great measure freed from pain, yet it returned when she plunged her hands into cold and warm water alternately, which she was often obliged to do; but I taught her, successfully, to repel it by percussion. In the end she informed me she could do this at pleasure. At her fifth visit, her shoulder and neck were entirely free from pain. I also succeeded in effecting perfect flexion and extension of the elbow-joint, almost without occasioning any pain; and, therefore, now considered the cure of this interesting case complete.

Case XXIII.—A lady, aged forty-nine, relict of a physician, consulted me, on the 16th February, 1822, for rheumatism, of uncommon severity, in the middle of the left shoulder-blade, all round the shoulder-joint, and at the elbow. She had complained for many months, and had, in vain, tried all the remedies that could be thought of, till her patience was entirely exhausted, and all hope of her condition ever being meliorated was extinguished. But what most annoyed the patient was the loss of motion in the shoulder-joint; her arm hung almost useless by her side; she could bring her hand forward a little, but that was owing to the motion of the shoulder-blade. She could not raise up her arm or put it backwards, because the shoulder-joint was as immovable as if anchylosed. Any motion, indeed, of which the arm was susceptible, either actively or passively, was solely on the shoulder-blade; for, after the most minute and patient examination, I could not tell whether anchylosis had taken place or not: all I could ascertain was, that there was no perceptible motion in the joint. In these circumstances, I could give no encouragement to my patient to expect a cure; at the same time I was anxious to make a trial, for ten days or so, of my mode of treating such cases, assuring her that, if no good was done, she should suffer no injury. She consented at once, desiring me to conduct myself according to my own judgment, without regarding her feelings, as she was determined to recover the use of her arm by all possible means.

A single point about the middle of the scapula, and another immediately below the elbow-joint, radial side of the arm, were so painful as scarcely to suffer being touched; the whole of the deltoid muscle, from origin to insertion, was also very tender; but, being compressible, these parts were quickly relieved from pain. To restore the motion of the joint was the difficulty, and for that purpose percussion alone was available. I put the joint as much upon the stretch as the patient's feelings would permit; then, fixing the scapula, I applied percussion above, behind, or in front of the joint, according to the position in which the arm was held. This process I alternated with attempts at rotation of the humerus, by bending the fore-arm, and converting it into a lever, which enabled me to apply percussion in a rotatory direction, with great effect. The operations were continued, with short intervals, for an hour every day. At the end of the first ten days, I could not say there was the slightest effect produced, except on the soft parts: in a fortnight, however, from the commencement of the operations, motion of the joint was palpable and unequivocal. Of this I satisfied the patient and all her family, to their great joy. Nothing was now wanting to a complete cure but perseverance in the mode of treatment I had begun: I therefore continued the operations every day for a fortnight longer, and every other day for two weeks more; when motion of the joint was completely restored, and has remained perfect ever since.

[To be continued.]

